# Mechanisms and therapeutic research progress in intestinal fibrosis

**DOI:** 10.3389/fmed.2024.1368977

**Published:** 2024-06-14

**Authors:** Yanjiang Liu, Tao Zhang, Kejian Pan, He Wei

**Affiliations:** ^1^School of Basic Medical Sciences, Chengdu Medical College, Chengdu, China; ^2^School of Bioscience and Technology, Chengdu Medical College, Chengdu, China

**Keywords:** intestinal fibrosis, pathological mechanisms, therapeutic research, epithelial-mesenchymal transition, cytokines and chemokines

## Abstract

Intestinal fibrosis is a common complication of chronic intestinal diseases with the characteristics of fibroblast proliferation and extracellular matrix deposition after chronic inflammation, leading to lumen narrowing, structural and functional damage to the intestines, and life inconvenience for the patients. However, anti-inflammatory drugs are currently generally not effective in overcoming intestinal fibrosis making surgery the main treatment method. The development of intestinal fibrosis is a slow process and its onset may be the result of the combined action of inflammatory cells, local cytokines, and intestinal stromal cells. The aim of this study is to elucidate the pathogenesis [e.g., extracellular matrix (ECM), cytokines and chemokines, epithelial-mesenchymal transition (EMT), differentiation of fibroblast to myofibroblast and intestinal microbiota] underlying the development of intestinal fibrosis and to explore therapeutic advances (such as regulating ECM, cytokines, chemokines, EMT, differentiation of fibroblast to myofibroblast and targeting TGF-*β*) based on the pathogenesis in order to gain new insights into the prevention and treatment of intestinal fibrosis.

## 1 Introduction

Fibrosis is a pathological physiological process in which the body responds to damage caused by harmful substances, such as physical, chemical, and mechanical injuries, infections, and autoimmune reactions, which can occur in various types of tissue injuries, particularly in chronic inflammatory diseases. The formation of fibrosis is due to the accumulation of extracellular matrix components, e.g., collagen and fibronectin, and is also an important stage in the repair of organs and tissues (1, 2). However, if the stimulus of fibrogenesis persists or manifests itself abnormally, tissue fibrosis and scarring may occur, which may even lead to organ dysfunction (3).

It is well-known that the formation of intestinal fibrosis is associated with inflammatory bowel disease (IBD), collagenous colitis, radiation enteropathy, eosinophilic enteropathy, cystic fibrosis, and other diseases (4–6). IBD is a chronic inflammatory condition with multiple potential causes, whereas radiation enteropathy is a specific type of intestinal damage caused by radiation therapy, eosinophilic enteropathy involves eosinophilic infiltration causing inflammation in the gastrointestinal tract and cystic fibrosis is a genetic disorder affecting both the respiratory and digestive systems due to mutations in the CFTR gene. Among these diseases, IBD, including Crohn’s disease (CD) and ulcerative colitis (UC), which is characterized by relapsing and chronic inflammation, and is associated with a 1.4 to 2.2-fold increased risk of colorectal cancer, is the main cause of intestinal fibrosis (7–10). Intestinal fibrosis is one of the most threatening complications of CD, affecting more than 50% of patients, and can progress from fibrotic strictures to penetrating or stenotic lesions. UC has long been considered a mucosal disease with no or minimal fibrosis. However, recent studies have shown that fibrosis can also be detectable in acute and chronic UC and in all sections of the colon, which correlates with the severity of the inflammation (11).

**Figure 1:**
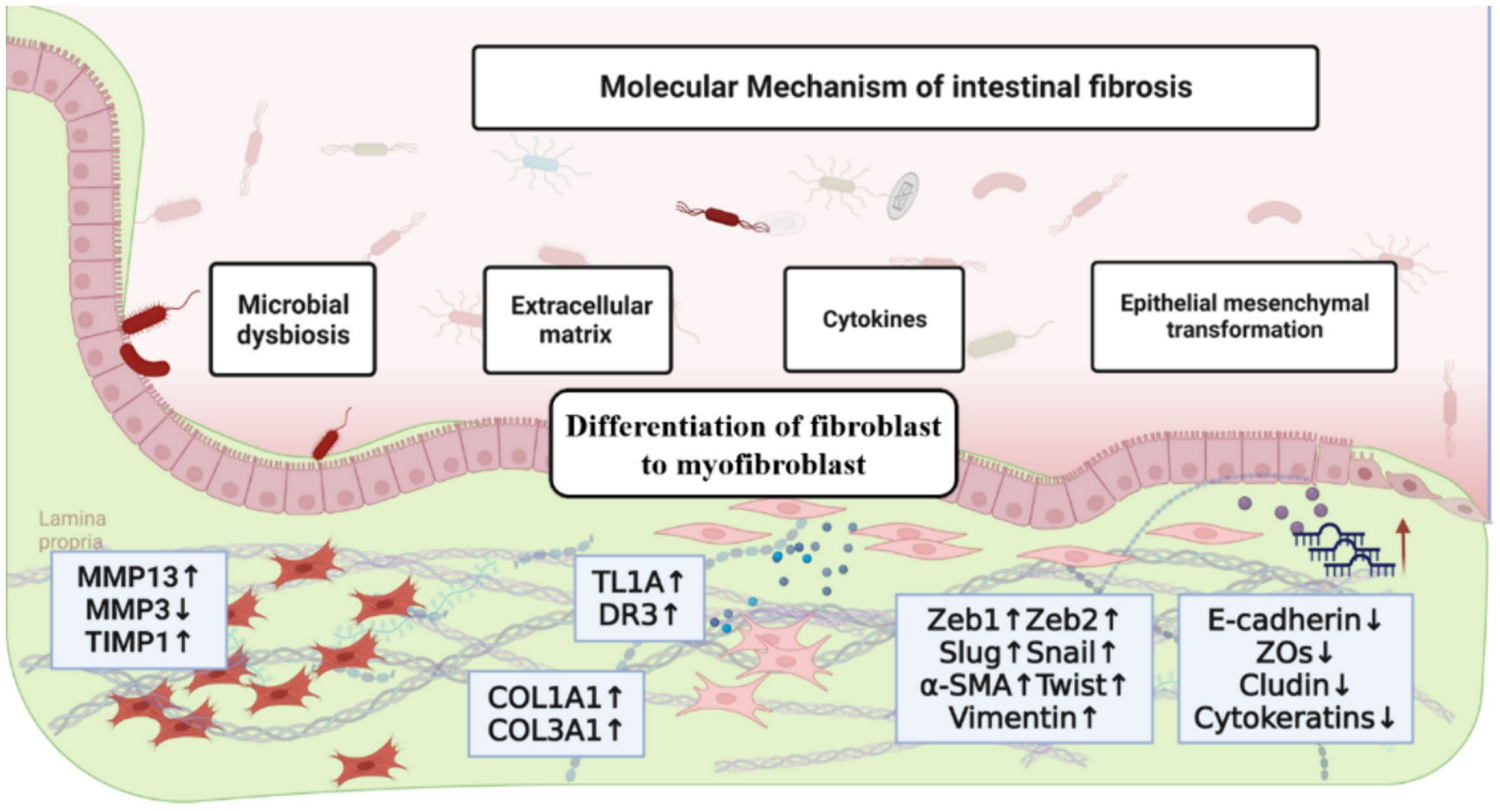
The molecular mechanism of intestinal fibrosis, including the influence of extracellular matrix, cytokines and chemokines, epithelial-mesenchymal transition, differentiation of fibroblast to myofibroblast and intestinal microbiota.

The imbalance of fibrotic regulation caused by the continuous irritation of intestinal inflammation is one of the typical features of intestinal fibrosis. In this context, the lumen diameter gradually decreases, resulting in intestinal stenosis, which can easily cause intestinal obstruction and even endanger the patient’s life (10, 12). Although the treatment of various gastrointestinal diseases has improved significantly due to the rapid development of medical technology and diagnostics, the formation of intestinal fibrosis is still high. In addition, pathological mechanisms play a crucial role in understanding and treating intestinal fibrosis. In this context, this study reviewed the progress of recent development of strategies for the treatment of intestinal fibrosis on the basis of elucidating the pathogenesis of intestinal fibrosis (Figure 1).

## 2 The pathogenesis of intestinal fibrosis

### 2.1 Extracellular matrix

The development of organ fibrosis is closely associated to the formation and activation of fibroblasts and the resulting deposition of extracellular matrix (ECM). The ECM is a complex network of proteins, elastin, hyaluronic acid, collagen and polysaccharides that surrounds and supports cells within tissues and organs and plays a crucial role in maintaining tissue integrity, providing mechanical support, and enabling various cellular functions (13, 14). Intestinal fibrosis occurs when there is an imbalance between the production and degradation of ECM components (15). It is reported that hyaluronic acid, one of the high molecular weight polymers under normal conditions, could be cleaved into low molecular weight fragments during excessive inflammatory actions, which promotes fibroblast proliferation and myofibroblast differentiation, thereby accelerating the fibrosis process (16). In addition, the low molecular weight fragments of hyaluronic acid help recruit immune cells to the site of inflammation, leading to the release of various inflammatory mediators and growth factors that further trigger fibrosis.

During the fibrosis formation process, antifibrotic and various profibrotic factors play a regulatory role, with the regulation of tissue inhibitors of metalloproteinases (TIMPs) and matrix metalloproteinases (MMPs) being of great importance. MMPs can degrade most of the extracellular matrix, such as MMP-1, MMP-9, MMP13, etc., while TIMPs can regulate the activity of MMPs. An imbalance in the MMPs/TIMPs system can lead to abnormal ECM deposition, contributing to the development of intestinal fibrosis (17, 18). It was found that MMP3 expression decreased in the fibrotic strictures of the intestinal mucosa in patients with CD, while TIMP1 expression was increased (19). Furthermore, researchers have observed significant upregulation of MMP13 in colon biopsies from the constricted areas compared to non-constricted areas in CD patients (20).

### 2.2 Cytokines and chemokines

Cytokines and chemokines secreted by immune cells and nonimmune cells act as coordinators of persistent inflammatory microenvironments, including interleukins (ILs), transforming growth factor-alpha (TNF-*α*), TGF-*β*, platelet-derived growth factor, insulin-like growth factor-1 and epidermal growth factor (21). The profibrotic effects result from the proliferation of fibroblasts caused by various growth factors and cytokines and provide a plausible avenue for new therapeutic targets in fibrotic diseases. Among these, ILs, TNF-*α* and TGF-*β* were the mostly widely studied. ILs involved in intestinal fibrosis generally include IL-11, IL-33, IL-34, IL-17, IL-36, etc., in which IL-11, a member of the IL-6 family, is considered a profibrotic cytokine that secreted by stromal cells and epithelial cells during tissue injury. Schäfer et al. found that transgenic expression of fibroblast-specific IL-11 or IL-11 injection in mice leads to cardiac and renal fibrosis and organ failure, while genetic deletion of IL-11RA can prevent the disease. Their experimental data illustrate that IL11 is a promising therapeutic target for IBD, particularly in the context of resistance to TNF therapy (22). Milara et al. reported that IL-11 and IL-11Rα are overexpressed in the pulmonary arteries of patients with idiopathic pulmonary fibrosis, which contributes to pulmonary artery remodeling and regulation of pulmonary arterial hypertension. Their work elucidates the role of IL-11 and IL-11Rα as driving factors in pulmonary arterial hypertension, suggesting their involvement in the process of pulmonary arterial remodeling (23). IL-33, a member of the IL-1 family that passively released upon cell damage and necrosis, is also involved in the fibrosis formation process. It was reported that IL-33 is closely associated with fibrotic progression in pediatric CD of the ileum due to increased epithelial expression (24). Remarkably, a recent study demonstrated a novel association between the IL-33/ST2 signaling pathway and intestinal dysbiosis in intestinal fibrosis. The authors observed that adherent invasive *Escherichia coli* (AIEC) colonization induced ST2 expression in the intestinal epithelium, thereby enhancing the IL-33/ST2 signaling pathway and ultimately promoting intestinal fibrosis. Their findings contribute to the expanding array of research investigating the underlying processes of fibrostenosis in CD, suggesting that eosinophils and mediators originating from eosinophils could serve as promising therapeutic targets for CD-associated strictures (25). Furthermore, IL-34 shows significantly increased expression in the damaged intestine of patients with CD and UC, which plays a crucial role in mediating crosstalk between immune cells and stromal cells and influences the production of fibrotic molecules by promoting signaling pathway activation (26). Another study reported that IL-34 stimulates the expression and secretion of COL1A1 and COL3A1 collagen by fibroblasts via a p38 MAPK-dependent mechanism, thereby promoting fibrogenesis (27).

Tumor necrosis factor-like ligand 1A (TL1A), a member of the TNF family, interacts with death receptor-3 (DR3) to form the TL1A/DR3 costimulatory system, while abnormal TL1A/DR3 signaling is involved in chronic inflammation and fibrogenesis formation process, and may contribute to the development of colitis-associated inflammation and fibrosis through the regulation of immune responses or inflammatory factors (28, 29). TGF-*β* has three subtypes, namely TGF-*β1*, TGF-*β2*, and TGF-*β3*, which play a crucial role in cellular development, proliferation and differentiation. They are the main regulatory factors driving organ fibrosis and play an important role in tissue fibrosis processes (30, 31). Smad proteins, the downstream proteins and signaling transducers in the TGF-*β* pathway, can form the TGF-*β*/Smad signaling pathway with TGF-*β*. Phosphorylated type II TGF-*β* receptor and TGF-*β* ligand combine to form heterodimeric complexes, which can recruit and activate type I receptors, promoting the activation and phosphorylation of downstream Smad2/Smad3 proteins. Smad4 can bind to P-Smad2/3 to form a complex, which then enters the cell nucleus to regulate relevant target genes (32, 33). It was reported that normal intestinal myofibroblasts primarily release TGF-*β3*, while myofibroblasts in ulcerative colitis express both TGF-*β1* and TGF-*β3*. In fibroblast cultures derived from CD fibrotic tissues, the expression of TGF-*β3* is significantly reduced, but the release of TGF-*β2* is enhanced (32, 34). Furthermore, it was also discovered that TGF-*β1* selectively enhances the production of collagen protein in human intestinal smooth muscle cells *in vitro* and plays an important role as an inflammatory mediator in the pathogenesis of intestinal fibrosis (35). Berger et al. showed that abrogation of TGF-*β* expression in T cells was completely able to disrupt T/B cell homeostasis and induce T cell-mediated inflammatory lesions in various organs, including the intestine (36). If the termination of wound healing due to various factors is delayed, TGF-*β* will persist in its overexpression, further fueling tissue fibrosis. Additionally, TGF-*β* plays a role in converting fibroblasts into myofibroblasts, which actively contribute to the deposition of ECM (37). Moreover, TGF-*β* has the capability to stimulate fibroblasts to produce more TGF-*β* internally, thereby establishing an autocrine loop that significantly contributes to the progression of fibrosis (38).

### 2.3 Epithelial-mesenchymal transition

EMT is a biological process in which epithelial cells undergo a transformation into mesenchymal cells, which, on the other hand, are more migratory and have the ability to differentiate into various cell types such as fibroblasts, smooth muscle cells, and immune cells (39). In addition, the loss of cell polarity, the enhanced invasion abilities, the downregulation of epithelial markers such as adhesion molecules, tight junction proteins (E-cadherin, ZOs and claudin), and cytokeratins, and upregulation of mesenchymal markers, including *α*-smooth muscle actin (*α*-SMA), vimentin, fibronectin, collagen and the EMT-associated transcription factors (Twist, Snail, Slug and Zeb1/2) are also features of the mesenchymal phenotype (40). Notably, a mounting body of evidence has emerged in recent years, underscoring a profound association between EMT and the development of intestinal fibrosis. EMT emerges as a pivotal indicator of intestinal fibrosis, characterized by the loss of epithelial polarity and phenotype, accompanied by a consequential metamorphosis into mesenchymal cells. Noteworthy, abnormal expression of EMT-associated molecules has been observed within fibrotic lesions of patients afflicted with CD affecting the intestines (41). It was also reported that EMT is involved in fistula formation, an abnormal channel between two epithelial cells that is associated with fibrosis (42).

The process of EMT in intestinal fibrosis is regulated by various signaling pathways and other mediators. Wang et al. demonstrate that miR-21 is significantly upregulated in intestinal tissue of CD patients with fibrotic strictures, followed by reduced PTEN expression, increased expression of EMT markers and mTOR, and imbalance in MMP9 /TIMP1. Knocking down miR-21 can reduce EMT *in vitro* and inhibit TNBS-induced intestinal fibrosis *in vivo* by inhibiting EMT and balancing MMPs/TIMPs. Their findings indicate that inhibiting miR-21 with antagonists could be a potential new therapeutic target for the treatment of CD fibrosis complications (43). Ortiz-Masi et al. elucidated the connection between the increased recognition of EMT and the WNT2b/FZD4 interaction in the intestinal tissue of CD patients. WNT2b stimulation elicits *in vitro* EMT by instigating the induction of FZD4, thereby potentially orchestrating the transmural engagement that characterizes penetrating CD. A comprehensive comprehension of FZD4’s involvement in this intricate mechanism may pave the way for novel therapeutic strategies aimed at averting complications arising from CD based on their findings (44). Certain pro-inflammatory cytokines, e.g., IL-17A, have been shown to involve fibrotic properties and are associated with fibrosis in multiple organs, including the intestine. The findings presented by Zhang et al. underscore the involvement of the EMT pathway as a key mechanism employed by IL-17A to drive intestinal fibrosis in both clinical CD patients and in a model of TNBS-induced chronic colitis. These results shed light on the potential of targeting EMT as a promising strategy for the development of anti-fibrotic therapeutics, with the prospect of mitigating IL-17A-mediated effects (45). To assess the functional state of TGF-*β*1 signaling within inflamed tissues of individuals with inflammatory bowel disease (IBD) and investigate the potential amelioration of impaired epithelial adhere and junction in IBD through the inhibition of TGF-*β*1 signaling using small molecules, Ghorbaninejad et al. directed their attention toward the modulation of EMT pathways. Their findings revealed the activation of both canonical and non-canonical TGF-*β*1 signaling cascades in inflamed intestinal epithelial cells, leading to the induction of EMT and disruption of epithelial tight junctions, which serve as distinguishing features of IBD (46). In addition, mediators such as Toll-like receptor 4 are also involved in inducing EMT and promoting intestinal fibrosis (43, 47). In addition to EMT, endothelial-mesenchymal transition (EndoMT) has also been reported as a novel mechanism of fibrosis (48).

### 2.4 Differentiation of fibroblast to myofibroblast

It is worth noting that the differentiation of fibroblast to myofibroblast also plays a crucial role in the development of intestinal fibrosis as myofibroblasts contribute to tissue repair and wound healing by secreting collagen and other matrix proteins (49, 50). In pathological conditions, persistent micro-stimuli activation of epithelial cells leads to the production of numerous cytokines at the site of injury. Cytokines like transforming growth factor TGF-*β* and tumor necrosis factor TNF-*α* trigger fibroblast proliferation, migration, accumulation, and differentiation into myofibroblasts, forming fibroblastic foci at sites of progressive intestinal injury and repair (51). The appearance of fibroblastic foci is a key feature in intestinal pathogenesis, indicating fibrotic areas during active progression and serving as a histopathological marker for irreversible fibrosis. These foci are primarily composed of myofibroblasts expressing *α*-SMA (52, 53). Myofibroblasts differ in ultrastructure and function from fibroblasts and smooth muscle cells, secreting an abundance of matrix metalloproteinases that disrupt the tissue matrix balance. Fibroblasts can also synthesize gelatinase to degrade the basement membrane during migration, hampering tissue regeneration. Myofibroblasts release angiotensinogen, promoting lung epithelial cell apoptosis and affecting tissue repair and remodeling. The activation of fibroblasts into myofibroblasts by injured intestinal epithelial cells perpetuates lung epithelial cell damage, creating a harmful cycle that contributes to the development of intestinal fibrosis.

### 2.5 Intestinal microbiota

Intestinal microbiota, also known as gut microbiota, refers to the community of microorganisms that inhabit the human gastrointestinal tract and form a relatively stable ecosystem through interactions with the host. It is estimated that there are 10^10^–10^14^ bacteria in the human gut, consisting of approximately 500–1,500 different bacterial species. As the largest and most complex ecosystem in the human body, the gut microbiota and its thousands of metabolic products have a profound impact on various aspects of host physiological activities (32). There seems to be increasing evidence that the gut microbiota influences intestinal fibrosis. Numerous investigations have demonstrated that chronic infection with Salmonella leads to the manifestation of profound and enduring intestinal fibrosis in mice, concomitant with the elicitation of protease expression in macrophages and epithelial cells (54). Furthermore, it has been observed that fibrosis can be directly instigated by microbial derivatives, particularly constituents of the cellular wall. Moreover, the existence of fecal matter or anaerobic bacteria in the gastrointestinal tract has been identified as a potential trigger for intestinal fibrosis (55).

In a study conducted by Jacob et al., the investigation focused on elucidating the impact of diverse microbial communities and their interactions with TL1A on the development of fibrosis. Remarkably, the study revealed the complete elimination of the pro-fibrotic and inflammatory phenotype induced by TL1A overexpression in the absence of a resident microbiota. To further unravel the intricate dynamics, the authors orally administered fecal samples obtained from specific pathogen-free (SPF) mice and healthy human donors to germ-free (GF) wild-type and TL1A-transgenic (Tl1a-Tg) mice. The outcomes unequivocally demonstrated that colonization with SPF microbiota resulted in escalated collagen deposition and activation of fibroblasts within the intestines of TL1A-Tg mice. Conversely, under GF conditions, fibroblast migration and activation were notably diminished, underscoring the indispensable role of specific microbial populations in TL1A-mediated intestinal fibrosis and fibroblast activation (56). In another investigation by Zhao et al., it was discovered that pretreatment with antibiotics augmented the ability of mice to restore their intestinal microbiota subsequent to radiation exposure. Intriguingly, this pretreatment effectively curtailed lipopolysaccharide (LPS) levels and modulated the polarization of ileal macrophages by inhibiting the TLR4/MyD88/NF-*κB* signaling pathway. Moreover, downregulation of TGF-*β*1, phosphorylated Smad-3, and SMA protein levels, coupled with upregulation of E-cadherin protein expression, was observed. Consequently, these alterations culminated in a reduction of inflammation and prevention of intestinal fibrosis. Significantly, these beneficial effects translated into improved post-radiotherapy survival rates and ameliorated intestinal damage (57). In summary, the involvement of the gut microbiota in the pathogenesis of intestinal fibrosis can manifest through direct mechanisms or via inflammation-mediated processes. Consequently, modulation of the gut microbiota may represent a promising therapeutic avenue for the treatment of intestinal fibrosis.

## 3 Research progress in the treatment of intestinal fibrosis

As the understanding of the pathogenesis of intestinal fibrosis deepens, new research into appropriate treatments has also emerged. Although traditional treatments, e.g., antibiotics and surgery, are used for intestinal fibrosis, there are currently no clinically approved targeted antifibrotic drugs for it. The development of therapeutic drugs primarily focuses on the underlying mechanisms of intestinal fibrosis with the aim of improving patient outcomes. Some recent promising therapies on intestinal fibrosis were summarized in Table 1.

**Table 1:** Some recent promising therapies on intestinal fibrosis.

Methods	Predominant compounds	Reference
Regulating ECM	Sulfasalazine	60
	Pirfenidone	61
Regulating cytokines and chemokines	Peroxisome proliferator-activated receptor-gamma (PPAR-γ)	62
	Paeonia decoction	63
	IL36R	65
	Anti-Tl1a antibody or an IgG isotype control	66
	Naringenin	68
	Colitis 1 formula and mesalazine	69
Targeting TGF-*β*	Calycosin (CA)	74
	Pre-gum arabic (GA)	75
	Vitamin D	76
	Adiponectin	77
	baicalin	78
Regulating EMT	PPAR-γ modulator	82
	halofuginone	83
	recombinant human bone morphogenetic protein-7 (rhBMP-7)	84
Regulating the differentiation of fibroblast to myofibroblast	proteoglycans and THY1	85,86
	AXL	87
	miR-29	89–91

### 3.1 Regulating ECM

As mentioned above, intestinal fibrosis is primarily caused by an imbalance in ECM deposition and degradation, regulated by MMPs and TIMPs. Therefore, targeting ECM regulation with drugs can achieve certain therapeutic goals. It is reported that fibroblast activation protein (FAP) is involved in fibrosis by regulating ECM deposition. The inhibition of FAP could reduce the production of type I collagen and TIMP1, suggesting that targeting FAP can restore ECM homeostasis in fibrotic strictures of patients with CD (58). Furthermore, in a mouse model of chronic intestinal inflammation, increased transcription of plasminogen activator inhibitor-1 (PAI-1) was observed in active fibrotic lesions, promoting TNBS-induced intestinal fibrosis. Oral administration of a PAI-1 inhibitor in this mouse model upregulates MMP9 protein, reduces collagen accumulation, and inhibits fibrogenesis. Some drugs are already available for ECM modulation. Sulfasalazine, a molecule with anti-inflammatory activity, protects the colonic mucosa from TNBS-induced damage and inhibits intestinal fibrosis by regulating the balance of TIMP/MMP proteins and ECM degradation (59). Pirfenidone is an antifibrotic drug that treats intestinal fibrosis by inhibiting the proliferation of intestinal fibroblasts and suppressing collagen production (60).

### 3.2 Regulating cytokines and chemokines

Cytokines and chemokines play a crucial role in the development of intestinal fibrosis and are effective targets for the treatment of intestinal fibrosis. It has been found that peroxisome proliferator-activated receptor-gamma (PPAR-*γ*) has anti-inflammatory and antifibrotic effects in multiple organs and can downregulate the production of pro-inflammatory cytokines interleukins. In preliminary clinical trials and experimental models of intestinal fibrosis, PPAR-*γ* has shown improvement in the fibrotic formation process. Therefore, studying new molecules that can improve inflammation and fibrotic lesions as PPAR-*γ* agonists may be an effective treatment for preventing and treating IBD and intestinal fibrosis (61). It is also reported that paeonia decoction (a traditional Chinese herbal formula) can improve radiation-induced enteritis in C57BL/6 mice and its potential protective mechanism may be associated with reduced expression of interleukins, thereby alleviating inflammation and improving intestinal fibrosis (62). Additionally, IL-36 promotes the secretion of pro-fibrotic mediators, and the expression of MMP13 is regulated by IL36R signaling, while MMP13-deficient mice exhibit less fibrosis in a chronic dextran sodium sulfate (DSS) model, with reduced numbers of αSMA^+^ fibroblasts. Therefore, the inhibition of IL-36R signaling has been proposed as a potential therapeutic strategy for fibrotic diseases (20). In mice with DSS or TNBS-induced colitis, IL-36 induces the expression of fibrosis-related genes in regulatory fibroblasts. Remarkably, the reduction of chronic colitis and intestinal fibrosis could be achieved by inhibiting or knocking out the IL36R gene in mice and the fibrosis phenomenon could also be significantly reduced by injecting of antibodies against IL-36R (63).

It is believed that blocking TL1A may have potential therapeutic effects in intestinal fibrosis. Li et al. reported that a chronic colitis model was established by injecting CD4^+^CD45RB^high^ naive T cells obtained from either C57BL/6 wild type mice or LCK-CD2-Tl1a-GFP transgenic mice into recombinase activating gene-1-deficient mice via intraperitoneal injection. The colitis model mice were subjected to either prophylactic or therapeutic treatment using anti-Tl1a antibody or an IgG isotype control. Histopathological changes in colonic tissue were assessed using Hematoxylin and eosin staining, Masson’s trichrome staining, and sirius red staining. Additionally, immunohistochemical staining was performed to evaluate the expression levels of collagen I, collagen III, TIMP1, vimentin, α-SMA, and TGF-*β1*/Smad3. The findings revealed that inhibiting the activation of intestinal fibroblasts and reducing collagen synthesis through the use of anti-Tl1a antibody contributed to the alleviation of intestinal inflammation and fibrosis in the chronic colitis model induced by T cell transfer. This effect may be attributed to the inhibition of the TGF-β1/Smad3 signaling pathway (64).

Recent studies also indicate that naringenin can improve DSS-induced colitis and colonic fibrosis in mice. After intervention with naringenin, UC mice showed a significant increase in body weight, a significant decrease in the ratio of colon weight to length, and a significant reduction in levels of TNF-*α*, IL-1*β*, IL-6, and the degree of colonic fibrosis. This demonstrates that naringenin can alleviate intestinal inflammation, improve intestinal barrier function, and inhibit DSS-induced colitis fibrosis (65, 66). The combination of colitis 1 formula and mesalazine has been found to be more effective in treating ulcerative colitis than mesalazine alone. This combined treatment reduces levels of TNF-*α*, IL-6, and IL-8, regulates fibrosis-related growth factors, and lowers inflammatory factor levels to promote ulcer healing (67).

### 3.3 Targeting TGF-*β*

Targeted therapy against TGF-*β* is the most promising anti-fibrotic treatment approach, as it is the major molecular mediator of fibrosis, with TGF-*β*1 being the most important among them. It was reported that local activation of angiotensin II strongly stimulates the production of TGF-*β*1. Angiotensin II is the main effector of the renin-angiotensin system, and its activity is elevated in the colonic mucosa of patients with CD. Therefore, it is believed that angiotensin-converting enzyme (ACE) inhibitors and angiotensin II receptor antagonists may play a role in the process of intestinal fibrosis formation, for example, captopril (68–70). The cholesterol-lowering drug simvastatin can also regulate TGF-*β* and exert its effects. Additionally, direct targeting of drugs has significant effects (71).

In a study conducted by Liu et al., the potential antifibrotic properties of calycosin (CA) were examined using CCD-18Co cells, which are human intestinal fibroblasts induced by TGF-*β*1. The MTT method was employed to determine the optimal concentration of *CA.* Furthermore, the expression levels of α-smooth muscle actin (α-SMA), collagen I, and the TGF-*β*/Smad pathway were assessed using real-time polymerase chain reaction and western blot analysis. The experimental findings indicated that CA exhibited concentrations of 12.5, 25, and 50 μmol/L. Notably, CA displayed inhibitory effects on the expression of α-SMA and collagen I. Furthermore, CA demonstrated the ability to modulate the TGF-β/Smad signaling pathway. Specifically, CA hindered the expression of p-Smad2, p-Smad3, Smad4, and TGF-β1, while enhancing the expression of Smad7, thereby suppressing the TGF-β/Smad pathway. Consequently, CA exhibited promise in mitigating intestinal fibrosis through the inhibition of the TGF-*β*/Smad pathway (72).

Al-Araimi et al. reported that colitis was induced in C57BL/6 mice using DSS, followed by their transition to normal drinking water to monitor the recovery process. The mice were divided into two groups: the pre-gum arabic (GA) group received 140 g/L GA prior to colitis induction, while the post-GA group received GA after colitis induction. Body weight changes, disease activity index (DAI), and histological assessment were used to evaluate disease activity and recovery. The expression levels of proinflammatory, anti-inflammatory, and fibrotic markers were measured in the colonic tissues. During the recovery phase, the pre-GA group exhibited an increase in body weight without significant differences in DAI scores. Moreover, this group demonstrated lower histological colitis scores compared to the post-GA group, which exhibited higher DAI and histological scores during recovery. Notably, the pre-GA group displayed increased expression of proinflammatory markers during the recovery phase, while the expression of fibrotic markers, including TGF-*β*1 and procollagen I, was reduced. The reduction in fibrotic marker expression correlated with decreased collagen staining and increased proliferation of epithelial cells. The administration of GA exhibited protective and ameliorative effects on the severity of DSS-induced colitis, reducing colonic fibrosis and suppressing TGF-*β*1 expression (73).

Tao et al. reported that mice deficient in vitamin D were randomly assigned to two groups at the weaning stage (week 4) and provided either a vitamin D-deficient or vitamin D-sufficient diet. Intestinal fibrosis was induced by administering 2,4,6-trinitrobenzene sulfonic acid every 6 weeks, starting from week 8. At week 14, the levels of extracellular matrix (ECM) production and total collagen were assessed in the colon. Additionally, the TGF-*β*1/Smad3 signaling pathway was investigated in isolated colonic subepithelial myofibroblasts (SEMF). The expression of vitamin D receptor (VDR), α-SMA, and Collagen I was evaluated in both normal SEMF and VDR-null SEMF following exposure to TGF-*β*1 and/or 1,25(OH)2D3. The findings indicate that vitamin D has a preventive effect on intestinal fibrosis in mice with chronic colitis and vitamin D deficiency, and its mechanism may be related to the inhibition of the TGF-β1/Smad3 pathway through VDR induction in SEMF (74). Furthermore, the treatment of intestinal fibrosis with adiponectin, baicalin, and the spleen-strengthening medicine are also related TGF-*β* (75–77).

### 3.4 Regulating EMT

Targeting EMT is another treatment for intestinal fibrosis and Atractylenolide III (ATL-III) may inhibit TGF-β1-induced EMT in IEC-6 cells by activating the AMPK signaling pathway, thereby inhibiting intestinal fibrosis (78). PPAR-*γ* is a member of the nuclear hormone receptor superfamily and acts as a ligand-activated transcription factor with various roles in lipid metabolism, inflammation, cell proliferation, and fibrosis. Significant impairment of PPAR-*γ* expression has been observed in colonic epithelial cells of IBD patients, suggesting that disruption of PPAR-*γ* signaling may be a key step in the pathogenesis of IBD. PPAR-*γ* overexpression can prevent tissue fibrosis, while its deficiency can increase fibrosis (79). It was reported that a novel PPAR-γ modulator, GED-0507-34 Levo, can improve DSS-induced intestinal fibrosis by regulating EMT mediators and pro-fibrotic molecules (80). Recently, halofuginone was used to inhibit TGF-*β*1-induced EMT in IPEC-J2 cells through the eIF2α/SMAD signaling pathway, making them a potential anti-EMT drug for the treatment of intestinal fibrosis (81). Furthermore, there is also evidence that recombinant human bone morphogenetic protein-7 (rhBMP-7) has anti-fibrotic effects, as it can inhibit TGF-β1-induced EMT associated with intestinal fibrosis (82).

### 3.5 Regulating the differentiation of fibroblast to myofibroblast

Myofibroblasts primarily stem from resident fibroblasts within the tissue, but can also arise from epithelial and endothelial cells, as well as other mesenchymal precursors. Various factors shape their differentiation, including cytokines, growth factors, the composition and rigidity of the extracellular matrix, and cell surface molecules like proteoglycans and THY1, which is also the key point in the treatment of intestinal fibrosis by regulating the differentiation of fibroblast to myofibroblast (51, 83). Steiner et al. found that activation of the AXL pathway occurs in models of intestinal fibrosis. Blocking AXL signaling using the compound BGB324 effectively prevents matrix stiffness and TGF-β1-induced fibrosis in human colonic myofibroblasts. Moreover, suppressing AXL with BGB324 enhances the vulnerability of myofibroblasts to apoptosis. Additionally, treatment with BGB324 inhibits the expression of fibrotic genes and proteins induced by TGF-*β*1 in human intestinal organoids (84).

Numerous studies have highlighted the dual role of miR-29 in tumor progression, particularly emphasizing its significant inhibitory impact on fibrosis (85). For instance, Yuan R et al. demonstrated that miR-29a contained in exosomes derived from human adipose-derived mesenchymal stem cells (hADSCs) directly interacts with TGF-*β*2, effectively suppressing the TGF-*β*/Smad signaling pathway and consequently impeding the development of pathological scars (86). Moreover, lncRNA H19 has been identified as a regulator targeting miR-29b to modulate the behavior of oral mucosal fibroblasts (87). In a different study, Yang J et al. established an *in vitro* model of silicosis cells with manipulated levels of miR-29c and observed that miR-29c successfully impedes the SiO_2_-induced trans-differentiation of lung fibroblasts *in vitro* (88). The competitive binding of lncRNA TUG1 with miR-29c enhances the hypoxia-induced fibroblast-to-myofibroblast transition (FMT) in cardiac fibroblasts (89). Consequently, circHIPK3 and the miR-29 family present themselves as promising therapeutic targets for fibrosis that merit additional investigation.

## 4 Future perspective

Currently, our understanding of the pathological mechanisms of intestinal fibrosis is still limited. Further research is needed to investigate the pathogenesis of intestinal fibrosis, such as inflammation-mediated fibrosis, extracellular matrix remodeling, and cellular activation, in order to reveal its development and progression mechanisms. With a deeper understanding of the mechanisms underlying intestinal fibrosis, we can explore new therapeutic targets. Factors such as cytokines, signaling pathways, and cell types that play a role in the fibrosis process are worth further investigation (90). Exploring these targets may lead to new treatment strategies. Intestinal fibrosis may be driven by multiple different mechanisms, thus individual variations may exist. Developing personalized treatment strategies and selecting the optimal treatment methods based on the specific pathological mechanisms of each patient can help improve treatment outcomes and prognosis. Due to the complexity of intestinal fibrosis, a single treatment may not be sufficient to completely reverse fibrosis (91). Therefore, combining multiple treatment modalities such as medication, surgical intervention, and physical therapy may produce better treatment outcomes.

## 5 Conclusion

In summary, the ECM, cytokines, and epithelial-mesenchymal transition play a crucial role in the occurrence, development, and treatment of intestinal fibrosis. In recent years, significant progress has been made in understanding the mechanisms and transformations of intestinal fibrosis, providing a comprehensive understanding of its pathogenesis and new strategies for clinical treatment. To some extent, the symptoms of intestinal fibrosis can be controlled, and long-term remission is possible. Although research on the cellular and molecular mechanisms underlying the pathogenesis of intestinal fibrosis is still in its early stages, anti-fibrotic therapy remains a challenging clinical need. However, as our understanding of the pathogenesis of intestinal fibrosis continues to grow, the emergence of new anti-fibrotic treatments holds great promise in effectively controlling disease progression and alleviating patient suffering.

## Data availability statement

The original contributions presented in the study are included in the article/supplementary material, further inquiries can be directed to the corresponding authors.

## Author contributions

YL: Writing – original draft. TZ: Investigation, Supervision, Writing – review & editing. KP: Writing – review & editing. HW: Writing – review & editing.
